# Authentication of Cocoa Products Based on Profiling and Fingerprinting Approaches: Assessment of Geographical, Varietal, Agricultural and Processing Features

**DOI:** 10.3390/foods12163120

**Published:** 2023-08-20

**Authors:** Sonia Sentellas, Javier Saurina

**Affiliations:** 1 Department of Chemical Engineering and Analytical Chemistry, Universitat de Barcelona, Martí i Franquès 1-11, 08028 Barcelona, Spain; sonia.sentellas@ub.edu; 2 Research Institute in Food Nutrition and Food Safety, Universitat de Barcelona, Av. Prat de la Riba 171, Edifici Recerca (Gaudí), 08921 Santa Coloma de Gramenet, Spain; 3 Serra Húnter Fellow Programme, Generalitat de Catalunya, Via Laietana 2, 08003 Barcelona, Spain

**Keywords:** cocoa, chocolate, authentication, profiling, fingerprinting, chemometrics

## Abstract

Cocoa and its derivative products, especially chocolate, are highly appreciated by consumers for their exceptional organoleptic qualities, thus being often considered delicacies. They are also regarded as superfoods due to their nutritional and health properties. Cocoa is susceptible to adulteration to obtain illicit economic benefits, so strategies capable of authenticating its attributes are needed. Features such as cocoa variety, origin, fair trade, and organic production are increasingly important in our society, so they need to be guaranteed. Most of the methods dealing with food authentication rely on profiling and fingerprinting approaches. The compositional profiles of natural components –such as polyphenols, biogenic amines, amino acids, volatile organic compounds, and fatty acids– are the source of information to address these issues. As for fingerprinting, analytical techniques, such as chromatography, infrared, Raman, and mass spectrometry, generate rich fingerprints containing dozens of features to be used for discrimination purposes. In the two cases, the data generated are complex, so chemometric methods are usually applied to extract the underlying information. In this review, we present the state of the art of cocoa and chocolate authentication, highlighting the pros and cons of the different approaches. Besides, the relevance of the proposed methods in quality control and the novel trends for sample analysis are also discussed.

## 1. Introduction

Cocoa is a product of tropical origin, native to America, that comes from the seeds in the pods of the cocoa tree (*Theobroma cacao*). Cocoa consumption dates back more than 2500 years, and in the Mayan, Aztec, and Inca civilizations was used for its nutritional and medicinal properties [1,2]. The tree was considered divine, and the fruit was recognized as a gift from the gods. Theobroma means, in Greek, food of the gods. It was also used as currency, an element of commercial exchange, and a key piece in the preparation of food and drinks. Cocoa was introduced in Europe in the fifteenth century, although the former chocolate drink recipes were not palatable to most of the population due to its bitter taste. In the 19th century, cocoa became much more appetizing with the addition of sugar to the preparations, and an important chocolate industry emerged.

The cocoa tree grows in the tropical region, which extends between 10 and 20 degrees north and south of the equator in the so-called “cocoa belt”, corresponding to West Africa, Central and South America, and Asia [3]. It requires a warm climate with high temperatures and constant and regular rainfall. Today, the main producer countries are African, such as the Ivory Coast, Ghana, Cameroon, and Nigeria, which produce more than 60% of the cocoa consumed worldwide.

Although only three botanical varieties are commercially relevant, there are several cocoa types: Criollo, Forastero, and Trinitario [4]. Forastero cacao is the most common, robust, and productive variety, accounting ca. 70% of the world’s total, with Ivory Coast, Ghana, and Cameroon being the main producers. Organoleptically, Forastero is stronger, bitterer, more acidic, and more astringent than the other varieties, so, in general, it is less appreciated; multinational food companies use it to manufacture large quantities of more ordinary chocolates. An important sub-variety of Forastero cacao is Nacional (also called Arriba), cultivated mainly in Ecuador and some areas of Peru. Despite the genetic similarities, they differ significantly in the shape and size of the fruits, aroma, and flavor, the latter being much more refined. Criollo cocoa is of the highest quality and presents fruity aromas and medium intensities, with notes of nuts and slight bitterness. It is grown mainly in Mexico, Guatemala, Venezuela, Peru, Madagascar, and Nicaragua and barely represents 10% of world production. The Trinitario cacao plant is a hybrid of the Forastero and Criollo varieties to combine the resistance and productivity of Forastero with the fine aroma and flavor of Criollo, thus producing a flavorful grain that is easy to grow. This variety is popular in Trinidad, Sri Lanka, Papua-New Guinea, Cameroon, and Venezuela. Additional information on the influence of varieties and processing on organoleptic product characteristics can be found in the review by Kongor et al. and Afoakwa et al. [5,6].

Cocoa beans require a long and complex post-harvest process consisting of fermentation, drying, roasting, and grinding to obtain the cocoa powder used in the manufacturing of cocoa products. Further steps of mixing, conching, molding, and packaging, as well as blending with other ingredients such as cocoa butter, sugar, milk, almonds, hazelnuts, fruits, etc., are applied to produce chocolate bars, bonbons, and other products. In the initial processes, especially fermentation and roasting, numerous chemical transformations are produced to achieve the desired organoleptic features of flavor and aroma [7,8,9].

Cocoa is a great source of bioactive compounds, of which polyphenols, with multiple beneficial properties, such as anti-microbial, antineoplastic, or anti-inflammatory features, can be highlighted [7,10,11]. These activities are mainly associated with the ability of phenolic compounds to combat oxidative stress thanks to their great antioxidant and antiradical power. It has been reported that a diet rich in polyphenols, such as those present in cocoa, strengthens the immune system, decreases the risk of diabetes, and reduces the incidence of cardiovascular diseases, among others. Methylxanthines such as caffeine and theobromine are also important due to their mild stimulating activity of the central nervous system, increasing awareness, attention, and energy levels. They also have a diuretic effect, and caffeine, especially, is involved in lipid metabolism, so combined with physical activity, it can contribute to reducing body fat. Also, biogenic amines, mainly generated during the cocoa fermentative processes, deserve special attention. Serotonin, responsible for a sense of well-being and stress relief, is one of the most important due to its bioactivity. However, negatively, tyramine and histamine are other of the most abundant amines that can trigger migraine attacks, high blood pressure, and allergies. Flavor and aroma attributes come from the wide variety of volatile organic compounds present in cocoa. Regarding the mineral content, cocoa is rich in some essential trace elements necessary for the proper functioning of the body, among which magnesium, copper, potassium, and iron stand out. However, it is important to note that such health benefits are obtained when products with a cocoa content greater than 70% are consumed.

The sensory and health properties of cocoa-based products depend on cocoa origin, post-harvest practices, and manufacturing procedures [9,12,13]. As commented above, these products are considered delicacies and superfoods with multiple health benefits. In this sense, premium products claim some differential features that make them more exclusive, such as the variety of cocoa used, the country or region of origin, and other agricultural or socioeconomic factors (fair trade, organic production, etc.). These characteristics make them preferred by consumers and reach higher prices than their conventional counterparts. Unfortunately, this greater commercial and social interest also makes them more susceptible to fraudulent practices to increase the economic benefit.

Traditionally, the exclusivity of a product was renowned mainly in the world of wines and spirits, where astronomical prices can be paid for some very specific bottles. However, the fashion for selecting certain products has quickly spread to other markets, such as extra virgin olive oil, acorn-fed ham, etc., which are also highly appreciated by consumers. Indeed, protected designations of origin, quality labels, and other recognition of food quality have been created to acknowledge products such as fruits and vegetables, beer, vinegar, spices, tea, coffee, and cocoa and its derived products.

This article presents the state of the art of cocoa and chocolate authentication, discussing the approaches applied and highlighting their pros and cons. Compositional profiles and/or instrumental fingerprints containing embedded discriminant compound information are used to characterize, discriminate, and authenticate the samples under study. In any case, the complexity of the data prevents the direct interpretation of the information; thus, chemometric methods are usually needed for extracting the relevant data and drawing the proper conclusions. Besides, some thoughts on future trends and the implications on quality control and the detection of adulterations will also be given.

## 2. General Approach to Cocoa Authentication

Beyond bulk cocoa and derived products produced industrially without any special recognition of quality, some artisan products produced in small batches pursue more select features to achieve higher quality standards. In these cases, information about varietal characteristics, origin, manufacturing processes, and even some tasting notes is accessible to customers, who purchase their favorite products based on it. Ensuring or authenticating cocoa’s origin, variety, cultivation process, etc., is not trivial, and powerful strategies are needed to recognize these characteristics to prevent consumers from being deceived. It is not only an economic matter since high prices are paid for those exclusive products but there may be toxicological consequences because of the fraudulent addition of undeclared materials. Supposedly, cocoa products are susceptible to counterfeiting or adulteration of a diverse nature.

As with many other foods, adulteration of cocoa and chocolate occurs in different scenarios. In this regard, Perez et al. have published a very interesting review on the traceability, authenticity and sustainability of cocoa and chocolate products [14]. As stated, these issues continue to be an important challenge for the chocolate industry, and many issues remain to be resolved. In some cases, cocoa used as raw material can be substituted or mixed with cheaper cocoas of lower quality. In this sense, the highest quality Criollo cocoa can be adulterated by adding (or substituting) other varieties intended for bulk production. The correct processing of cocoa is also essential, especially during fermentation, in which certain qualities, flavors and aromas are conferred [5,6]. Thus, to recycle or reuse and not have to throw away any defective fermented batches, they can be concealed by mixing them with adequate amounts of other batches that are correct. Also, unexpected materials (e.g., flour, shells, sugars, fats, etc.) can be mixed to increase the mass of production at a lower cost. The shell case deserves special attention since, during the manufacturing process, it is impossible to remove this residue from the cocoa paste completely, and a small fraction is always present [15]. Indeed, the Codex Alimentarius Commission establishes a maximum of 5% shell and germ bud content in the non-fat dry matter obtained in cocoa manufacturing. The occurrence of higher amounts indicates intentional additions, careless processing, or lower-quality products. Apart from the economic fraud that higher amounts of shell waste represent, the physical, organoleptic, and nutritional features of cocoas and chocolates produced with this deficient raw material can be affected. The issue of fats in adulteration is also very relevant due to the addition of products of animal origin, such as lard or natural or synthetic cocoa-butter equivalents (CBEs). Although some of these fraudulent practices may be nutritionally relevant, generating problems of contaminants, allergies, cholesterolemia, hyperlipidemia, etc., the economic and cultural repercussions are even more remarkable, for example, with halal and kosher food issues.

Cocoa and chocolate authentication, like other foodstuffs, can be based on sensory testing by expert panelists. However, this strategy is expensive and is reserved for very specific cases. Indeed, it would be unfeasible for quality control and authentication of many samples. In addition, the panelists’ scores may show a certain dispersion due to inter-individual variability, fatigue, etc. Alternatively, the characterization of agri-food products based on analytical methods seems to be often more convenient [16].

One of the analytical approaches for food authentication, which has been used successfully in many cases, relies on DNA detection after amplification by polymerase chain reaction. This easy, fast, and cost-effective methodology exhibits excellent performance in selectivity and detection limits. In the case of cocoa, such an approach can be useful for dealing with some quality control aspects, such as distinguishing fine and bulk cocoa genotypes [17]. Another interesting fraud problem is detecting lard added intentionally to chocolate [18]. The bacterial population responsible for cocoa fermentation, related to geographical conditions, is very important in developing flavor features; this bacterial composition can also be studied by high-throughput sequencing technologies combined with DNA amplification [19]. However, the DNA amplification by the polymerase chain reaction from cocoa and chocolate samples is difficult, so the experimental conditions must be thoroughly investigated to obtain reliable procedures.

**Figure 1:**
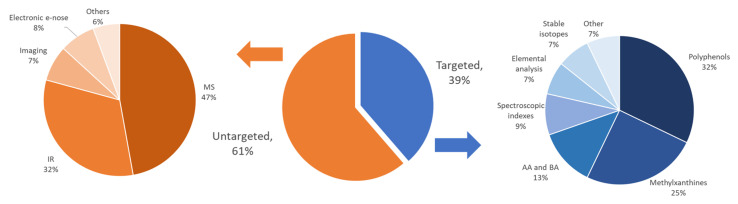
Distribution of fingerprinting (untargeted) and profiling (targeted) approaches for the characterization, classification and authentication of cocoa and chocolate samples. Acronyms: MS, Mass spectrometry; IR, Infrared spectroscopy; AA, Amino acids; BA, Biogenic amines.

Beyond the strategies to search for a specific marker of the supposed sample alterations, a more widely used option relies on -omics, commonly metabolomics, under both profiling and fingerprinting approaches. In a broad sense, metabolomics studies the whole metabolome, understood as the set of all metabolites/compounds of an organism or derived sample. This concept has a more limited scope in food chemistry, thus focusing on a list or family of compounds that could be potential descriptors or markers of the features under study. The metabolomic strategies used today are based on both profiling and fingerprinting approaches, the latter being the most exploited strategy in the characterization and authentication of cocoa products (Figure 1).

Regarding the first option, in the case of cocoa, the compositional profiles of naturally occurring compounds, such as polyphenols, biogenic amines, amino acids, volatile organic compounds and fatty acids, can be employed as the data. As for fingerprinting, complex instrumental signals (e.g., spectra or chromatograms) are used as the data without assuming anything about the possible compounds responsible for the generated signals. In a way, within these spectra or chromatograms, peaks/signals related to the chemistry of the samples can be recognized and used for descriptive purposes. In fingerprinting, rapid and simple analytical instruments, such as infrared and Raman spectroscopies, are sometimes the ideal choice. Liquid and gas chromatography with ultraviolet, fluorescence or mass spectrometric detection may also offer great opportunities to generate efficient fingerprints to tackle authentication issues. In any case, the adulteration can be reasonably detected if, within the set of analytes (or instrumental signals) under study, some appear at levels significantly higher (up-regulated) or lower (down-regulated) than in samples considered normal, meaning that adulterated samples do not fit the pattern of the authentic counterparts.

The compositional profiles and/or fingerprints may contain embedded discriminant information that can allow characterization, discrimination, and authentication of samples. However, the complexity of the data entails that this information is not evident. It is hidden and cannot be interpreted with a glance. Hence, in the course of authentication processes, chemometrics is essential to deal with the great amount of data provided by profiling and fingerprinting approaches and to try to extract the underlying information. The general flowchart follows different stages, beginning with exploratory studies as a first insight into the characteristics of the samples that also allow for the recognition of potential adulteration marker compounds or signals. Exploratory studies are unsupervised, meaning that they do not assume the existence of classes or distinctions. Such markers are rarely selective. That is, they are not only present (or absent) in the fake samples, but they are overexpressed or down-expressed in the counterfeit samples with respect to the authentic ones. This is often carried out by principal component analysis (PCA), multivariate descriptive statistics, and cluster analysis.

**Figure 2:**
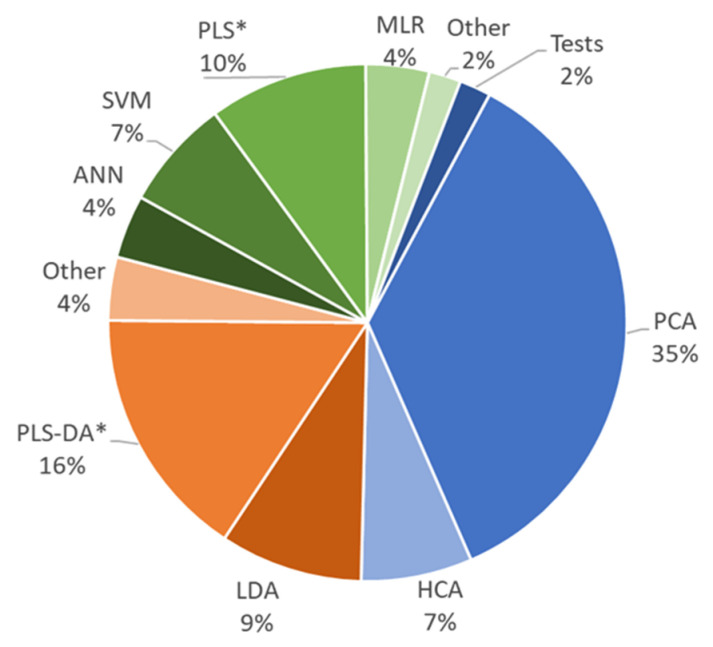
Chemometric methods used for the characterization, classification and authentication of cocoa and chocolate samples. Acronyms: PCA, Principal component analysis; HCA, Hierarchical cluster analysis; LDA, Linear discriminant analysis; PLS-DA, Partial least squares–discriminant analysis; ANN, Artificial neural networks; SVM, Support vector machine; PLS, Partial least squares (regression); MLR, Multiple linear regression. The asterisk means these PLS methods also include sequential and orthogonal modalities.

Furthermore, the significance of these markers can be verified statistically, thus corroborating that the altered samples differ, with a pre-established confidence level, from the authentic. The assignation of samples into predefined groups such as geographical origins, varieties, elaboration procedures as well as the discrimination among genuine versus fraudulent samples is currently tackled by classification methods such as Partial Least Squares-Discriminant Analysis (PLS-DA), Linear Discriminant Analysis (LDA), Soft Independent Modelling of Class Analogy (SIMCA), and others. Furthermore, in some cases, the adulteration extent can be quantified by multivariate calibration with Partial Least Squares (PLS) and related regression methods to determine the amount or percentage of adulterant illicitly added to a given cocoa or chocolate sample. Figure 2 shows a pie graph revealing that PCA is the most popular method in exploratory studies applied in 35% of the cases. For classification, the PLS-DA has been used more widely (including its S- and OPLS-DA variants) and applied to the 16% of the publications. Finally, the authors rely on the PLS (and its variants) to quantify adulterations since it has been used in 10% of the cases. However, non-linear modeling, with neural networks, SVM, etc., has also shown remarkable importance.

## 3. Fingerprinting-Based Authentication

Non-targeted strategies, where instrumental responses are used as fingerprints, catch the attention of researchers because of their simplicity and great analytical performance. Unlike the targeted analysis discussed in the next section, previous knowledge of the sample composition is not required, and exhaustive method development is unnecessary. Indeed, around 60% of the publications since 2015 dealing with cocoa or cocoa products authentication use this approach (Figure 1). Among the arsenal of instrumental techniques available, infrared spectroscopy (IR) and mass spectrometry (MS) are the preferred ones (Figure 1a). Other detection techniques, such as nuclear magnetic resonance (NMR), spectroscopies (UV and fluorescence), electroanalytical analyses, or electronic noses, were less exploited in this field [20,21,22,23,24,25].

IR spectroscopy is a vibrational technique that measures the interaction of the radiation in the IR region with the molecules. These interactions depend on the type of bonds (length and angle) of the analyzed components. Thus, information about the functional groups of the molecules can be extracted from the obtained spectrum. IR is, therefore, a good technique to be used for identification purposes. However, it can also be exploited in untargeted analysis considering the full spectrum without paying attention to the individual signals. For such a purpose, multivariate data analysis is required to extract the relevant information and discard noise or useless data. Both mid-IR (MIR, 2500–25,000 nm region) and near-IR (NIR, 800–2500 nm region) have been extensively applied to the authentication of foodstuffs in general, and cocoa in particular as has been reviewed by Castillejos-Mijango et al. and Teye et al., respectively [26,27]. It is important to note that the generated spectra often require extensive preprocessing to improve their quality and remove background noise, baseline drift, scattering, and other potential deficiencies that otherwise would hinder the analysis. Hence, smoothing, first derivative, second derivative, multiplicative scatter correction, mean centering, and standard normal variable is applied, often as a sequential combination of some of them.

These publications emphasize the potential of IR spectroscopy and the most successful chemometric techniques to characterize and detect adulterations. As stated before, determining whether a product is pure or has been adulterated and the percentage of adulteration is paramount to avoid fraudulent practices. Without looking for a specific adulterant, Santos et al. developed two IR-based (NIR and MIR) methods for determining cocoa solids, meaning the percentage of cocoa present in a chocolate sample [28]. First, spectroscopic data were normalized and subjected to PCA. Samples were clustered along PC1 according to cocoa content. Two calibration models to predict the percentage of cocoa were established, with mean prediction errors below 2%. Finally, they applied the models to 110 commercial chocolate samples finding that the percentage of cocoa in ca. 14% of the samples differs from the informed value. More specifically, the cocoa adulteration with peanut or carob flour or cocoa shell was also evaluated by untargeted analysis using IR spectra combined with different chemometric tools, namely PLS, SVM, and MCR-ALS [15,29,30,31]. In one of the examples, the percentage of cocoa shells was estimated using a simple and fast FT-NIR method combined with chemometrics [15]. The authors built an SVM regression model that allows the determination of the percentage of cocoa shells from 0 to 20% with errors around 2%, independently of the geographical origin, harvesting period, or roasting process. Differentiation among cocoa varieties, clones, and geographical origin was also performed by treating NIR spectra with several chemometric tools [32,33,34,35,36,37]. Among these studies, it is worth mentioning the work by Cruz-Tirado et al. because, unlike most reported studies, they analyzed cocoa bean hybrids directly, i.e., without a previous sample grounding treatment [33]. NIR hyperspectral imaging (NIR-HIS) allows the authors to extract not only spectral but also spatial information; the data treatment by PLS-DA and SVM acceptably distinguished between five different Brazilian cocoa bean hybrids [33], with SVM being the method with higher predictive performance, with prediction errors ranging from 3.8 to 23%. In this area, the growing importance of portable and handheld equipment to perform field measurements for cost-effective, sensitive, in situ and real-time food characterization and to verify its authenticity and traceability [38]. Although these devices do not have the spectroscopic quality of the more expensive benchmark instruments, combined with highly effective procedures for data pre-treatment and chemometric methods for data analysis, they provide high performance regarding correct classification and authentication. For example, the product quality, highly dependent on the harvesting and production (fermentation and roasting) processes, was assessed by Anydoho and co-workers using portable NIR spectroscopy [39,40]. NIR spectra recorded in the range 900 to 1700 nm were preprocessed using various treatments, including first and second derivative, multiplicative scatter correction, mean centering, and standard normal variable, with multiplicative scatter correction being the most suitable option. The resulting data were analyzed with classification methods such as PLS-DA, random forest (RF), and K-nearest neighbors (KNN) differentiated organic and conventional harvesting processes with calibration and prediction rates around 90% [39]. Similarly, fermentation features strongly related to the quality of the cocoa beans were satisfactorily predicted using NIR and multivariate analyses (LDA, SVM, and PLS) [40].

Also concerning the IR region, Raman spectroscopy is an excellent source of information widely used in authentication issues. In addition, it is available in a handheld format for in situ studies. Vargas Jentzsch et al. used this technology to distinguish Nacional cacao from clone CCN-51 [41]. Both varieties were differentiated with a performance rate greater than 90% using SVM after data processing by baseline correction, normalization, and noise filtering.

Table 1 summarizes these and other relevant applications of IR techniques for the characterization and authentication of cocoa and cocoa products.

**Table 1:** Studies addressing the characterization and authentication of cocoa and cocoa products based on IR techniques.

Sample Type	Analytical Technique	Data Analysis	The Objective of the Study	Reference
Cocoa powder	Vis/NIR	PLS	Determination of cocoa shell content	[29]
Cocoa nibs	FT-NIR	SVM	Determination of cocoa shell content	[15]
Cacao nibs	NIR	CAFS; NBC	Discrimination of Amazonian cacao nibs from six cacao clones	[32]
Chocolate	FTIR conventional techniques	PLS	Quantification of BAs (spermidine, tryptamine, cadaverine, and tyramine), polyphenols and DPPH activity. Differentiation of cocoa varieties used for production	[42]
Chocolate	NIR and MIR	PCA; PLS	Determination of cocoa solid content and detection of adulterations	[28]
Cocoa bean	NIR	LDA; SVM; PLS	Determination of product quality based on fermentation features and other parameters (pH and moisture content).	[40]
Chocolate	NIR PTR-ToF-MS; SPME-GC-MS; LC; LC-MS	S-PLS; O-PLS	Classification into sensorial poles	[43]
Chocolate powder	NIR hyperspectral imaging	MCR-ALS	Detection of chocolate powder adulteration with peanut flour	[30]
Cocoa beans	NIR	PCA; RF; K-NN; LDA; PLS-DA	Differentiation between organic and conventional beans	[39]
Cocoa beans	NIR-hyperspectral imaging	PLS-DA; SVM	Discrimination of five cocoa bean hybrids	[33]
Cocoa beans	NIR	LDA; SVM	Classification according to geographical origin (Africa)	[34]
Cocoa bean shells	NIR; ATR-FT-IR (NIR and MIR); ICP-OES	PCA; PLS-DA	Geographical origin authentication	[35]
Cocoa powder	NIR	PCA; PLS-DA; PLS	Detection and quantitation of chocolate powder adulteration with carob flour	[31]
Cocoa beans	NIR	PCA	Classification and characterization of cocoa varieties	[36]
Cocoa beans	NIR; colorimetric sensor e-nose	ELM; SVM; LDA; k-NN	Determination of fermentation level (fully, partially, and non-fermented) as quality marker	[44]
Cocoa beans	FT-NIR	LDA; SVM; PCA	Authentication of cocoa varieties	[37]

IR: infrared spectroscopy; NIR: near-IR; MIR: mid-IR; FT-IR: Fourier transform-IR; ATR: attenuated total reflectance; PTR-ToF-MS: proton transfer reaction-time of flight-mass spectrometry; SPME-GC-MS: solid phase micro extraction-gas chromatography-mass spectroscopy; LC-MS: Liquid chromatography-mass spectrometry; ICP-OES: coupled plasma-optical emission spectroscopy; SVM: Support vector machine; CASF: covering array feature selection; NBC: naive Bayes classifier; PLS-R: partial least squares regression; PCA: principal component analysis; PLS-DA: partial least squares-discriminant analysis; LDA: linear discriminant analysis; S-PLS: sequential-PLS; O-PLS: orthogonal-PLS; MCR-ALS: multivariate curve resolution—alternating least squares; RF: random forest; K-NN: K-nearest neighbors; ELM: extreme machine learning.

As commented above, mass spectrometry (MS), as a standalone technique or combined with conventional separation techniques, including LC, GC, or supercritical fluid chromatography (SFC), is extensively used for untargeted characterization and authentication of cocoa products (Table 2). Both low- and high-resolution mass spectrometry (LRMS and HRMS) generate fingerprints highly rich in chemical information. However, HRMS is generally preferred, especially when previous separation techniques are not considered. For instance, matrix-assisted laser desorption/ionization coupled to a time-of-flight mass spectrometer (MALDI-TOF) was used for a preliminary untargeted analysis to identify the main components of the studied chocolate matrices [45]. Nevertheless, the data obtained was not used directly for discrimination purposes but served to select target compounds for further determination by LC-MS/MS (MRM mode), which, combined with PCA, discriminate chocolates according to the geographical origin of the cocoa beans used [46]. In another example, Deuscher et al. exploited direct injection mass spectrometry (DIMS) for food authentication, evaluating the organoleptic differences of dark chocolates based on their volatilome [47]. In this case, 206 chocolate samples, classified by expert panelists into four different “sensory poles,” were analyzed by proton transfer reaction MS (PTR-MS), and the data obtained submitted to PLS-DA modeling, obtaining a good differentiation according to the organoleptic attributes (with a prediction rate of 97% attained with the proposed methodology) [47]. Rapid evaporative ionization mass spectrometry (REIMS) is an emerging technique to directly characterize samples by MS without any (or with minimal) sample pretreatment. This is the case of the study performed by Chang et al. in which fingerprints obtained by REIMS were treated by means of different chemometric tools (HCA, OPLS-DA, and CSVM) to detect adulterations of cocoa butter (CB) with CB equivalents (CBE) [48]. The results were excellent, with classification accuracies of 100% and adulteration levels detected down to 10%.

Regarding LRMS as a standalone technique, the work of Scavarda et al. can be highlighted [49]. In this interesting study, headspace-solid phase microextraction-MS-E-nose (HS-SPME-MS-E-nose) demonstrated a high throughput alternative to HS-SPME-GC-MS for discriminating smoky from non-smoky cocoa. The authors treated the obtained fingerprints with PLS-DA and soft independent modeling by class analogy (SIMCA), achieving prediction abilities higher than 90%. Particularly, high sensitivities (correct identification of in-class samples) were obtained with the SIMCA models [49].

Despite the discriminatory potential of MS, the hyphenation of a complementary separation technique gives the untargeted analysis an additional dimension to be exploited in the generation of fingerprints. GC coupled to MS (GC-MS) is typically used to determine volatile compounds. Thus the volatile metabolome of cocoa or derived products can be used for classification and authentication. This technique, combined mainly with PCA, has been used to distinguish the geographic origin [50,51,52,53] as well as product quality [54] or cocoa genotype [50]. Similarly, LC-MS is extensively used for obtaining characteristic fingerprints that, after being treated with the appropriate chemometric methodology, enable the discrimination of products based on geographical origin, fermentation stage or cultivar [55,56,57,58]. Apart from these more common applications, an untargeted LC-MS approach was also established for determining the adulteration of cocoa powder with chicory and carob and soy flours [59]. Natural cocoa powder and simulated adulterated samples were analyzed with the optimized LC-MS (in positive and negative modes). From 1132 variables initially extracted from the LC-MS fingerprints, 58 variables were selected as potential markers to distinguish between natural and adulterated samples based on the PCA and PLS-DA models [59].

**Table 2:** Studies addressing the characterization and authentication of cocoa and cocoa products based on mass spectrometry techniques.

Sample Type	Family of Compounds *	Analytical Techniques Applied	Data Analysis	Objective of the Study	Reference
Cocoa powder		HPLC-MS (TOF)	PCA; PLS-DA	Determination of adulteration with carob flour, soy flour and chicory	[59]
Chocolate	CBE; triacylglycerol; theobromine and caffeine; sugars	UHPSFC-HRMS		Determination of quality and authenticity	[60]
Cocoa butter	Cocoa butter	REIMS	HCA, OPLS-DA; OCSVM	Determination of adulteration with CBE	[48]
Cocoa beans		LC-MS	Neural networks	Determination of fermentation and processing state and geographical origin	[55]
Chocolate		PTR-TOF-MS; SPME-GC-MS; LC; LC-MS; NIR	S and O-PLS	Classification into sensorial poles	[43]
Cocoa		LC-MS	PCA; LDA	Classification according to geographical origin	[56]
Cocoa		HS-SPME-MS-e-nose	SIMCA; PLS-DA	Detection of smoky off-flavor (inappropriate artificial drying)	[49]
Cocoa bean shells		HPLC-PDA-MS/MS (Qtrap)	PCA; Kruskal–Wallis and Wilcoxon tests; Pairwise Spearman’s correlation	Classification according to cultivar and geographical origin	[57]
Chocolates		MALDI-TOF (preliminary study).	PCA	Differentiation of geographical origin	[45]
Dark chocolate	Fat content, fatty acids, and volatiles.	HS-SPME/GC-MS	HCA; PCA	Differentiation and authentication of geographical origin and cocoa bean genotype	[50]
Unroasted cocoa beans	Volatiles	HS-SPME-GC-MS	PCA	Differentiation of geographical origin	[52]
Cocoa liquor and chocolate	Non-volatiles and volatiles	HS-SPME-GC-MS; UPLC-HRMS	PCA	Differentiation of geographical origin (Ecuador vs. West Africa)	[61]
Cocoa products		HPLC-qTOF-MS	S-PLS	Determination of shell content	[62]
Cocoa bean shells	Volatiles	HS-SPME/GC-qMS	PCA	Classification according to quality and origin	[51]
Cocoa	Volatiles	GC×GC-TOF-MS	FDR: LDA; PLS-DA	Classification according to the origin	[53]
Chocolate	Phenolic composition	LC-LRMS	PCA; PLS-DA	Discrimination of chocolate sensory groups (four) and quality sensorial poles	[63]
Dark chocolate	Volatiles	Direct injection MS	PLS-DA	Classification into sensory categories (four)	[47]
Cocoa beans (fermented/ unfermented)	Triacylglycerols	HPLC-HRMS	PCA; HCA	Differentiation according to fermentation status and geographical origin	[64]
Cocoa beans	Carbohydrates	HILIC-ESI-TOF-MS	Multivariate analysis	Differences according to fermentation status	[65]
Cocoa	Volatiles	GCxGC-MS	PLS-DA; MFA	Classification according to origin and processing steps	[66]
Cocoa	Volatiles	GCxGC-MS	PCA; fisher ratios; linear regression trees	Classification and discrimination according to geographical origin and manufacturing step and sensory-quality characterization	[67]
Dark chocolate	Volatiles	PTR-TOF-MS	PCA; PLS-DA	Classification according to botanical and geographical origin; relation of brand-related processing	[68]
Cocoa beans	Volatiles	HS-SPME-GC-MS	PCA	Identification of markers for bean origin/quality	[54]
Chocolate		MALDI-TOF	HCA	Determination of cocoa content	[69]
Chocolates	Non-volatile profiles	FI-ESI-MS	PCA; k-NN	Authentication of geographical origin	[70]
Cocoa beans		LC-TOF-MS	PLS-DA	Discrimination according to growing regions	[71]

* When the untargeted and targeted analysis is performed, only the untargeted analysis is considered here. CBE: cocoa butter equivalent; LC: liquid chromatography; HPLC: high performance liquid chromatography; UHPLC: ultra-high performance liquid chromatography; HILIC: hydrophilic interaction liquid chromatography; GC: gas chromatography; UHPSFC: ultrahigh performance supercritical fluid chromatography; PDA: photo diode array; MS: mass spectrometry; HRMS: high-resolution MS; LRMS: low-resolution MS; ESI: electrospray; HS: head space; SPME: solid phase microextraction; TOF: time of flight; PTR-MS: proton-transfer-reaction-mass spectrometry; MALDI: matrix-assisted laser desorption/ionization; REIMS: rapid evaporative ionization MS; HCA: hierarchical cluster analysis; PLS-DA: partial least squares-discriminant analysis; OPLS-DA: orthogonal PLS-DA; OCSVM: one- class support vector Machine; S-PLS: sequential-PLS; O-PLS: orthogonal-PLS; PCA: principal component analysis; LDA: linear discriminant analysis; SIMCA: soft independent modelling of class analogies; k-NN: k-nearest neighbors; MFA: multiple factor analysis; FDR: Fisher discriminant ratios.

To address the classification and authentication of cocoa products following an untargeted approach, LC, or GC—mostly combined with MS—are the techniques of choice; these platforms take advantage of the separation power of the chromatography and the selectivity and structural information provided by MS. However, LC or GC methods are not limited to these couplings. For instance, GC with electronic noses (E-nose) generated odorant fingerprints capable of distinguishing the different organoleptic properties to classify dark chocolates in the four preestablished sensory categories [21]. In this study, using correspondence analysis and hierarchical analysis, the authors identified 21 key odorants as key aroma compounds of dark chocolates. Also, a GC E-nose methodology has been demonstrated to be suitable to discriminate cocoa liquors according to their geographical origin using PCA and discriminant factor analysis (DFA) [23].

## 4. Profiling-Based Authentication

The profiling approach is based on quantifying the concentrations of various compounds naturally present in cocoa or chocolate to be used as the source of information [14]. The compositional profiles obtained can be studied using descriptive statistics and chemometrics to address their authenticity. Table 3 summarizes the most relevant data from recent contributions published on this topic. Most papers usually focus on a given family of compounds. However, data from several analyte sources, determined by one or several analytical methods, can also be treated together by the data fusion strategies outlined in Figure 1. The most relevant results, organized by families of compounds, are discussed as follows.

### 4.1. Phenolic Compounds

According to the pie chart in Figure 1, polyphenols are the most exploited source of information for the authentication of cocoa and chocolate, and they are used in more than 30% of profiling cases. Compared to other plants, cocoa is one of the richest matrices in polyphenols, highlighting for its quantitative importance some flavanols (epicatechin, catechin, and procyanidins B2 and C1), phenolic acids (gallic and 3,4-dihydroxybenzoic acids). In addition, a wide range of other phenolic compounds is present at concentrations of the order of magnitude of mg kg^−1^ and below, which, despite being less relevant quantitatively, are highly influential as potential markers or descriptors of cocoa qualities or varieties [14]. Apart from their qualitative importance as descriptors, the role of polyphenols, especially the majority ones, as healthy compounds are well-known [72]; their antioxidant and anti-radical properties give cocoa and black chocolate a superfood status [7,8,10].

The most common way to determine phenolic compounds is by liquid chromatography with UV spectroscopic (HPLC-UV) detection or coupled to mass spectrometry (HPLC-MS) [73]. Analytes are separated in reverse phase mode, generally with C18 stationary phases, using elution gradients generated from an aqueous solution of an organic acid (formic or acetic) and an organic solvent (acetonitrile or methanol). The elution gradients are normally optimized for each situation based on the compounds of interest. Depending on the family of analytes and their structural characteristics, different detection wavelengths in UV-vis spectroscopy are used: at 275–280 nm, hydroxyphenolic acids and flavanols are well detected, which, as mentioned above, are the most abundant compounds in cocoa; hydroxycinnamic acids such as caffeic, coumaric and derivatives absorb around 320–330 nm, and some minor flavonols are monitored at 370–380 nm [72]. Mass spectrometry (MS), especially in multiple reaction monitoring (MRM) modes, is much more sensitive and selective than UV-visible and allows the determination of minority cocoa polyphenols at concentrations below 1 mg Kg^−1^ [72]. Although the compositional profiles are determined as a source of information, global information is also of great interest.

**Table 3:** Studies addressing the characterization and authentication of cocoa and cocoa products based on targeted analysis.

Sample Type	Compounds	Analytical Technique	Data Analysis Method	Objective of the Study	Reference
Cocoa beans under fermentation	Phenolic compounds and methylxanthines	UHPLC, FC, DPPH, Total anthocyanins, pH, acidity	Polynomial correlation	Assessment of the evolution of bioactive compounds throughout the fermentation. Total polyphenols and antioxidant capacity decrease during fermentation while the anthocyanin increased	[74]
Chocolate	Triacylglycerol; theobromine and caffeine; sugars	UHPSFC-HRMS		Estimation of fat content and chocolate solids	[60]
Cacao beans	Epicatechin, catechin; procyanidins; theobromine, caffeine	HPLC-UV	PCA	Study of two clones (introduced and regional) in Colombia. Geographic origin, harvest conditions, year of harvest	[75]
Chocolate	Bioactive amines; phenolic compounds; antioxidant capacity	FTIR; DPPH	PLS	Multivariate prediction of individual BAs (spermidine, tyramine, tryptamine, etc.), polyphenols, and DPPH index in cocoa varieties	[42]
Ruby, dark, milk and white chocolate	Flavanols; proanthocyanins; methylxanthines; biogenic amines;	UHPLC-HRMS	PCA	Characterization of Ruby chocolate.	[76]
Chocolate	Polyphenols, organic acids	LC-MS	S and O-PLS	Assessment of sensorial poles	[43]
Cocoa bean shells (by-product)	Polyphenolic and methylxanthines profile; TPC; TFC; TTC	HPLC-UV; FC; DPPH	PCA	Assessment of origin and variety of cocoa bean shells as a waste to revalorize	[77]
Cocoa bean shells (by-product)	Phenolic compounds and methylxanthines	HPLC-UV-MS/MS (Qtrap)		Study of Forastero, Trinitario, Criollo and Nacional varieties collected in several Central and South American and African	[57]
Chocolates	Polyphenols	LC-MS/MS	PCA	Assessment of geographical origin	[45]
Dark chocolate	Fat content, fatty acids, and volatiles. TP; anthocyanin; methylxanthine and catechin	HS-SPME/GC-MS;	HCA; PCA	Assessment of geographical origin and genotype. Found markers up-expressed in the different classes	[50]
Cacao and cocoa products	Theobromine, caffeine, phenolics and flavonoids	DART/TOF-MS	PCA	Assessment of quality and authenticity	[78]
Cocoa beans	Methylxanthines, TPC, flavanols, proanthocyanins	HPLC-UV	PCA	Characterization of Nacional cacao produced in three regions of Ecuador	[79]
Cocoa (fermented, unfermented)	Theobromine, caffeine	Square-wave voltammetry		Comparison of fermented vs. unfermented samples	[80]
Chocolate	Theobromine, catechin, epicatechin, and caffeine	HPLC-UV	PCA, HCA	Study of artisanal and fine chocolate	[81]
Cocoa beans	Amino acids	GC-MS, derivatization with BSTFA		Evolution of amino acids with fermentation and roasting	[82]
Cocoa beans	^2^H, ^13^C, ^15^N, ^18^O; %C, %N, %O, %H	IRMS; H-NMR		Assessment of countries and varieties	[83]
Chocolates	Elemental analysis	ICP-MS	CART	Assessment of origin. Significant in Ba, Cd, Mo, and Sr	[84]
Cocoa bean shells	Elemental analysis	ICP-OES	PCA; PLS-DA	Assessment of geographical origin	[35]
Unroasted and roasted beans	Biogenic Amines; polyphenols; aroma compounds	HPLC-UV; GC-MS		Study of origin and effect of roasting. Temperature affects volatiles and biogenic amines	[85]
Bolivian Amazonian Cacao	Theobromine, catechin, total antioxidant capacity and TPC	HPLC-UV; ABTS; FRAP		Comparison before and after fermentation	[86]
Cocoa from Venezuela	Catechins and procyanidins	HPLC-UV		Study of geographical origin and post-harvest processes	[87]
Cocoa beans	Triacylglycerols	HPLC-MS		Identification of >80 volatiles	[88]
Cocoa products	Catechin and epicatechin	MALDI-MS imaging		Determination of cocoa content	[89]
Organic chocolate	Elemental analysis (38 elements)	ICP-MS	PCA; PLS-DA	Discrimination of organic vs. conventional chocolate	[90]
Cocoa beans	Bioactive amines, TPC, anthocyanins; ABTS	FC; ABTS; HPLC-FLD	PCA; HCA	Compositional changes during cocoa beans fermentation	[91]
Cocoa beans	Oligopeptide pattern	UHPLC-MS	PCA	Patterns of unfermented, under-fermented, and well-fermented cocoa beans. Geographical origin	[92]
Cocoa beans and products	Elemental analysis (Ag, As, Ba, Be, Bi, Ca, Cd, Co, Cr, Cs, Cu, Fe, Ga, Hg, K, Li, Mg, Mn, Na, Ni, P, Rb, Se, Sr, Th, Tl, U, Y and Zn)	ICP-MS	PCA; DA	Traceability of cocoas from East and West Africa, Asia and Central and South America	[93]
Cocoa derivatives	Biogenic amines	LC-ELSD	PCA; HCA	Assessment of conventional, organic and fair-trade cocoa derivatives	[94]
Cocoa-based products	Biogenic amines	HPLC-UV	ANOVA	Discrimination of conventional vs. organic products. Organic samples contain lower amine levels	[95]
Chocolate	Cocoa solids, TPC, methylxanthines, phenolics (catechin and epicatechin), antioxidant capacity	FC; DPPH; HPLC-UV	PCA; MLR	Determination of cocoa percentage in chocolates	[96]
Cocoa powder, chocolate, and supplements	Flavanol and procyanidins	UHPLC-FLD	PCA	Discrimination of cocoa products with the flavanol and procyanidins profile	[97]
Cocoa beans	Isotopic profile (^2^H, ^13^C, ^15^N, ^18^O, ^34^S)	IRMS	CDA	Discrimination of geographical origin from Africa, Asia, Central and South America	[98]
Fermented cocoa beans	Multi-element stable isotope ratios (^2^H/^1^H, ^13^C/^12^C, ^15^N/^14^N, ^18^O/^16^O); %C, %N	IRMS	PCA; PLS-DA	Discrimination of geographical and varietal origin	[99]

ABTS: 2,2′-azino-bis(3-ethylbenzothiazoline-6-sulfonic acid assay; ANOVA: Analysis of Variance; BAs: Biogenic amines; BSTFA: N,O-bis(trimethylsilyl)trifluoroacetamide; CA: cluster analysis; CART: Classification And Regression Tree; CDA: Canonical Discriminant Analysis; DA: Discriminant Analysis; DART: Direct analysis in real time; DPPH: 2,2-Diphenyl-1-picrylhydrazyl assay; ELSD: evaporative light scattering detector; FC: Folin-Ciocalteu assay; FLD: Fluorescence detector; FRAP: Ferric Reducing Antioxidant Power assay; FTIR: Fourier-Transform Infrared; GC: gas chromatography; HCA: Hierarchical cluster analysis; HRMS: high-resolution MS; HS: head space; ICP: Inductively-Coupled Plasma; IRMS: Isotope-ratio mass spectrometry; MALDI: Matrix Assisted Laser Desorption/Ionization; MLR: Multiple linear Regression; NMR: Nuclear magnetic resonance; MS/MS: Tandem Mass Spectrometry OES: Optical Emission Spectroscopy; O-PLS: orthogonal-PLS; PCA: principal component analysis; PLS: partial least squares; PLS-DA: partial least squares-discriminant analysis; S-PLS: sequential-PLS; TOF: time of flight; TFC: total flavonoid content; TPC: Total Phenolic Content; TTC: total tannin content; UHPLC: ultra-high performance liquid chromatography; UHPSFC: ultrahigh performance supercritical fluid chromatography.

In this context, the overall area recorded at 280 nm provides a very valuable estimate of the total content of phenolic compounds, with results often expressed in mg kg^−1^ of gallic acid equivalents (GAE) [100]. Complementary overall information is also gained through spectrophotometric indices of total polyphenols (Folin-Ciocalteu method, FC in GAE), global reducing power (Ferric reducing antioxidant power, FRAP), or antiradical capacity (DPPH), with values for the latter expressed as Trolox equivalent antioxidant capacity (TEAC) closely related to the overall polyphenol content [101]. In authentication studies, this information by itself is generally insufficient since these indexes have no selective ranges regarding varieties or origins, but it is of great help as a complement to the profile of phenolic compounds. In this sense, ca. 10% of characterization or authentication applications based on polyphenolic composition also include spectroscopic outcomes (antioxidant and antiradical) data to improve the descriptions.

As mentioned before, the profile of phenolic compounds is the most widely used source of information for the characterization of cocoa (see details of the latter publications in Table 3). Some papers will be discussed in more detail below as illustrative examples. Using the content of catechins, some procyanidins, and xanthines, as well as unknown but quantitatively relevant peaks recorded by HPLC-UV, Agudelo et al. explored the possibility of distinguishing among Colombian samples collected in different years belonging to introduced and autochthonous clones [75]. PCA results showed some structuration according to these features, but some class overlapping also occurred. In another study, about fifty phenolic compounds were determined by LC-MS/MS in MRM mode, and the compositional data were applied to the differentiation of cocoas based on origin [45]. The PCA results revealed that the samples from Ecuador were richer in bioactive compounds.

Regarding analytical innovations, a non-chromatographic rapid method by Direct analysis in real time (DART) time-of-flight MS (DART-TOF-MS) was used to quantify some phenolic acids, flavonoids, and xanthines [78]. As a proof of concept, the method was applied to cocoa powder and instant cocoa beverages, observing a good separation between both types of samples. In another representative example, the concentrations of xanthines, total polyphenols, flavan-3-ols monomers (catechin and epicatechin), and main proanthocyanidins (procyanidins B2 and C1) in beans of Nacional cacao produced in three regions of Ecuador, and PCA results showed some patterns regarding these characteristics [79]. In addition to these works, Table 3 reports similar studies based on phenolic content as a source of information [81,87,99].

Beyond cocoa and chocolate, various studies focus on cocoa bean shells as by-products rich in bioactive compounds to be considered for their revalorization in the framework of a circular economy. In the publication by Rojo-Poveda et al., the classification of cocoa bean shells of Forastero, Trinitario, Criollo, and Nacional varieties collected in several Central and South American and African countries was studied based on the global phenolic content provided by several spectrophotometric methods (total phenolics, flavonoids, tannins, and DPPH antiradical capacity) and the profiles of polyphenolic compounds determined by HPLC-UV [77]. PCA maps revealed that the compositional characteristics of the shells depended to a great extent on their provenance. According to varieties, the samples also showed remarkable clustering among classes. In another study by the same authors, they used LC-UV-MS/MS for similar purposes, focusing part of their efforts to identify potentially relevant compounds from the different categories [57].

### 4.2. Methylxantines

Theobromine and caffeine are the two major alkaloids of the methylxanthine family, present in cocoa and dark chocolate in ca. 1.5–3% and 0.2–0.8% (*m*/*m*), respectively [8,14]. As they are only two variables and without very outstanding differences in the compositional data, the capability of theobromine and caffeine to classify or authenticate the cocoa-related samples is limited. Minor alkaloids, such as theophylline or theacrine, have scarcely been considered for descriptive purposes. Although they are not specific markers of qualities or varieties, in general, Forastero cocoa is richer in theobromine than the other cultivars, while Criollo stands out for its higher caffeine content.

The pie graph (Figure 1) shows that methylxanthines have been used in 25% of the references, although they are considered alone but combined with the polyphenolic composition. Hence, theobromine and caffeine provide valuable complementary information to reinforce the quality and performance of the characterizations. Besides, such data is easily available since polyphenols and alkaloids are often determined simultaneously through reversed-phase liquid chromatography (RP-HPLC), with theobromine and caffeine eluting in the range of polyphenols with high and medium polarity, respectively. Their detection by UV spectroscopy is carried out at 280 nm. In most of the papers commented on above, phenolic compounds and methylxanthines are jointly quantified, so we will not make additional considerations in this regard.

### 4.3. Amino Acids, Peptides and Biogenic Amines

Amino acids (AAs) combined in the form of peptides and proteins are abundant in a wide variety of food. However, in their free form, they occur at low levels in most foods, except for fermented products, for which the content of free AAs may increase dramatically because of microbial degradation of proteins [102]. In this sense, as with wine, beer, cheese, and other dairy products, some types of tea, etc., free amino acid levels in cocoa are relevant, with leucine, isoleucine, valine, threonine, lysine, tyrosine being the most abundant, present in concentrations of the order of 1 to 100 mg Kg^−1^.

In parallel to AAs, biogenic amines (BAs) formed mainly from the decarboxylation of their corresponding precursors are also found in cocoa and related matrices. Values of the order of magnitude of 10 mg Kg^−1^ are given in some studies, highlighting tyramine, tryptamine, putrescine, phenylethylamine, histidine, and serotonin. The stimulant activity associated with cocoa consumption is attributed to amines, such as serotonin, and other well-known bioactives, such as theobromine or caffeine. The ability to trigger migraine episodes in sensitive individuals is also due to BAs such as tyramine or phenylethylamine. These compounds may have some value as descriptors of quality and hygienic conditions of foods in general [102], particularly cocoa.

Only a few amino acids and BAs have chromophore or fluorophore groups suitable for UV and fluorescence (FLD) detection at low concentrations, so they must be derivatized to improve the method sensitivity. Dansyl chloride is used mainly as a derivatizing agent in cocoa characterization because it confers excellent detection features, reacts with primary and secondary amino groups, the reaction is fast and clean (i.e., the formation of side products is limited) under mild conditions, and the derivatives are stable for several days. Dansyl derivatives are monitored at 320–330 nm in UV and at λ_exc_ 330 nm and λ_em_ 410 nm in FLD [102]. Besides, from the separation point of view, derivatization substantially improves the chromatographic performance, being highly recommended even when working with MS detection. Indeed, dansyl derivatives of amino acids and biogenic amines can be separated efficiently by reversed phase under experimental conditions similar to polyphenols and alkaloids using proper elution gradients based on increasing methanol or acetonitrile percentage [102].

The publications on the authentication of cocoa and chocolate based on the composition of amino compounds are, for the moment, limited, involving approximately 15% of the published works. However, we consider that these compounds are excellent quality descriptors and especially sensitive to fermentative processes; hence, they can be useful in the development of new studies.

Del Rosario et al. determined the AAs in cocoa beans subjected to different fermentative and roasting processes, in this case almost by GC-MS and derivatization with N,O-bis(trimethylsilyl)trifluoroacetamide (BSTFA) [82]. The authors revealed that, for Criollo cocoa produced in different locations in Venezuela, the increase in free AAs with fermentation reached the maximum values on the third day, highlighting that although lysine, leucine, alanine, proline, or phenylalanine are among the most abundant species, their concentrations vary significantly depending on the origin. After roasting, the values decreased remarkably (2 or 3 times lower). Restuccia and coworkers published several papers highlighting the role of BAs as quality descriptors, being useful even to differentiate between conventionally and organically grown cocoa. They identified several amines, including cadaverine, serotonin, histamine, spermidine, spermine, tyramine, putrescine, and β-phenylethylamine, using HPLC with evaporative light scattering detection or UV [94,95], concluding that the organic samples contain lower concentrations of these amines, thus suggesting their importance in the quality of the samples. Also related to the amino compounds, the oligopeptide profile determined by LC-MS was used to establish patterns related to the fermentation processes and the origins of the products [92]. Finally, Tuenter et al. developed a UHPLC-HRMS method to simultaneously quantify phenolic compounds, xanthines, and amino compounds to discriminate between dark, ruby, and white chocolate [76]. Samples belonging to each class were clustered and separated from the others by PCA.

### 4.4. Elemental Composition and Isotopic Profile

Elemental and isotopic composition are successful sources of information for food authentication, especially concerning their geographical origin. This type of data has been used in approx. 7% of the profiling publications. Plants obtain inorganic nutrients from the soil, and these minerals are distributed through their vegetative parts, reaching the fruit. Hence, cocoa beans’ elemental and isotopic composition depends on the proportion of assimilable elements in the soil [54]. Besides, it is highly influenced by the type of agriculture (conventional or organic) practised since the addition of natural or chemical fertilizers, and other phytosanitary products will also play a role in the inorganic profile of cocoa [86].

Atomic emission techniques using inductively coupled plasma with optical (ICP-OES) or spectrometric (ICP-MS) detection are the most reasonable option to carry out elemental composition determination [103]. The optical detection is robust and compatible with many types of extracts without a noticeable matrix effect, although it has difficulties detecting elements at concentrations below 0.1 mg L^−1^. MS detection has better quantification limits, up to two orders of magnitude lower. However, it is more sensitive to the composition of the matrix and the presence of organic solvents in the extracts analyzed.

Vanderschueren et al. used the multi-elemental composition determined by ICP-MS to establish the geographical origin of the chocolates and quantify the presence of toxic heavy metals such as Cd and Pb, whose content is limited by European legislation [56]. Regarding toxicological aspects, they found some samples that exceeded the permitted levels. Regarding the study of origins, significant differences were found in Ba, Cd, Mo, and Sr levels. Then, successful classification and regression tree (CART) models were established on continent classes. In another study by Junior et al., 38 elements were determined by ICP-MS as a source of analytical information to differentiate conventional and organic cocoa [90]. It was observed that some essential elements, such as Fe, Zn, and Mg, are encountered at higher concentrations in the organic samples. Exploratory PCA models and classifications by linear discriminant analysis were also established, which provided higher than 80% classification rates. The elemental composition was also used to assess the geographic traceability of cocoas from East and West Africa, Asia, and Central and South America using ICP-MS [93]. The discriminant models provided excellent results for classifying the samples according to the subcontinents.

An isotopic profile signature is an excellent approach for finding class markers, especially those related to the origin. Elements such as carbon, nitrogen, oxygen, etc., as the constituents of some organic molecules or inorganic isotopic rations are often considered. For instance, ^2^H, ^13^C, ^15^N, ^18^O, and ^34^S data obtained by isotope ratio mass spectrometer (IRMS) were used to assess cocoa beans from different origins, including samples from Africa, Asia, and Central and South America [98]. Classification models as a function of origin by Canonical Discriminant Analysis offered an acceptable classification rate of ca. 80%. Conclusions indicated that ^13^C was associated with climate and altitude features, ^18^O dealt with rains, and ^15^N and ^34^S depended on geology and fertilization practices. In another study, ^2^H/^1^H, ^13^C/^12^C, ^15^N/^14^N, ^18^O/^16^O ratios and the percentage of C and N were analyzed by exploratory PCA and supervised PLS-DA to try to find patterns concerning the geographical origin of cocoa beans [99]. The stable isotope profile and the percentages of C, O, H, and N were also used by Bindereif et al. to establish the geographical origins of cocoa beans [83].

### 4.5. Triglycerides and Volatile Organic Compounds (VOCs)

Cocoa beans are very rich in fat, approximately 50 to 60% of their weight corresponds to triglycerides, the most abundant fatty acids being stearic, oleic, and palmitic (each ca. 25 to 40%, depending on the cases); linoleic occurs at much lower percentages (ca. 3%) [48,81]. During the production of cocoa paste and further cocoa powder, a large fat percentage is removed by extrusion, giving rise to cocoa butter [14]. However, the residual fat in cocoa paste is still remarkable since it contains around 10 or 20% triglycerides. Along with them, small amounts of other volatile compounds of low polarity are also co-retained that will contribute to defining the aroma of cocoa and chocolates. Triglyceride and VOC profiles have been used as descriptors for the characterization, classification, and authentication of a wide range of food samples, although, so far, their potential for the study of cocoa products has been scarcely studied and as shown in Figure 1, only ca. 5% of cocoa authentication studies rely on these components. However, given the possibilities with other matrices, we think this approximation will gain importance for future studies.

The determination of triglycerides, related fatty acids, and other volatile compounds is carried out by gas chromatography with mass spectrometry detection (GC-MS) or flame ionization (GC-FID). Capillary columns with optimized temperature gradients for each type of analyte family are used to achieve good resolution. In some cases, GC × CG is used to try to increase the ability to separate a greater number of compounds by taking advantage of the orthogonality of the separation. Information can be incremented when high-performance detection techniques such as time-of-flight mass spectrometry (TOF-MS) are used. This approximation was used by Cordero et al. to generate a metabolomic profile of volatile compounds from cocoa samples from different origins [64]. Although the possibilities of fingerprinting in the characterization and classification of the samples were studied, a list was established based on Variable Importance for Projection (VIP) values from PLS-DA with the 20 most characteristic chemical markers to differentiate the samples of raw and roasted cocoa. Using the same instrumental platform, this working group previously investigated the volatile profile of cocoa samples corresponding to different stages of processing (raw, roasted, steamed, and ground beans) and produced in various countries (Mexico, Ecuador, Venezuela, Colombia, Java, Trinidad, and Sao Tome) [68]. In the targeted study, up to 130 compounds were identified that allowed good discrimination of samples by PCA and supervised multivariate methods. In another work, the compositional profiles of several dozen volatile compounds, including esters, alcohols, aldehydes, ketones, pyrazines, terpenes, and short-chain acids, among others, were determined by GC-MS [52]. Remarkable differences in the distribution of VOCs between unroasted and roasted cocoa beans were revealed. In addition, the profile of volatiles treated with PCA or LDA provide reasonable discrimination between continental origins (America, Africa, and Asia). Based on an alternative novel technique such as ultrahigh performance supercritical fluid chromatography coupled to high-resolution tandem mass spectrometry (UHPSFC-HRMS), Rektorisova et al. determined multiple analytical parameters from a single run method, including cocoa butter equivalents (CBEs), milk fat content, non-fat cocoa, and sugars profile to quality cocoa indexes [60]. The volatile profile determined by headspace-solid phase microextraction GC-MS (HS-SPME/GC-MS) was also used to study different genotypes of cocoa beans from Latin America [48]. PCA and HCA were used to characterize the samples, finding markers from North America (e.g., tetramethylpyrazine, trimethylpyrazine, benzaldehyde, and furfural) or South America (e.g., 2-methylpropanoic acid, 2,3-butanediol, 2-nonanone, and limonene), and other from regional genotypes (e.g., 2-phenylethyl acetate, 3-methyl-butanal, and cinnamaldehyde).

### 4.6. Data Fusion: Combined Data Sets

Data generated by different methods can be combined through fusion approaches to enrich the data sets and enhance descriptive performance. The papers discussed here rely on the low-level mode, i.e., simply incorporating the data from each analysis in the same matrix for a joint study with no previous pretreatment.

A recent paper assessed the changes in the composition of bioactive compounds throughout the fermentative process of cacao grown in the Peruvian Amazon, combining the composition of the most relevant phenolic compounds and methylxanthines with data from Folin-Ciocalteu, DPPH antiradical activity, total anthocyanins, pH and acidity [74]. The authors concluded that the total polyphenols and antioxidant capacity decrease during fermentation while the anthocyanin content increases slightly. Spizzirri and coworkers applied various chromatographic methods to determine polyphenols and methylxanthines, biogenic amines, and volatile species to assess the compositional features of fermented and roasted cocoa beans [85]. The authors encountered some variation patterns depending on the characteristics of the roasting processes, with temperature being a remarkable factor in the profiles of volatiles and biogenic amines. Another comprehensive study involving several families of components (phenolics and biogenic amines) plus antioxidant indexes and complementary data addressed the effect of the fermentation process [91]. Finally, the study by Mandrile et al. explored the power of various analytical techniques, including diffuse reflectance near-IR spectroscopy (NIRS), attenuated total reflectance mid-IR spectroscopy (ATR-FT-IR), and ICP-OES for the characterization of cocoa bean shells from different geographical origins [35]. Infrared techniques provided PCA score maps with considerable overlap between sample types.

Regarding the multi-element study, the PCA showed trends in the distribution of the samples from Africa and America. Subsequent PLS-DA classification models allowed the detection of samples of African origin with a reasonable classification rate. Fused data models were also established to try to improve the descriptive quality.

## 5. Conclusions

This review highlights the significance of cocoa and chocolate authentication since they are food matrices quite vulnerable to adulteration. Therefore, efficient analytical methods are needed for its control to ensure qualities such as botanical varieties, geographical origins, or manufacturing processes. In this sense, recognizing the finest variety of Criollo cocoa, differentiating them from bulk cocoa, or establishing regional origin are remarkable issues already addressed and resolved quite successfully.

The publications discussed in this manuscript are based on untargeted and targeted strategies using instrumental and chemical data for sample authentication. In general, it is necessary to have a representative set of perfectly known samples that is analyzed to assess the patterns and sample behavior. This process is carried out typically by applying chemometric methods of multivariate analysis. Among them, unsupervised exploratory methods reveal the natural trends of the samples. Principal Component Analysis is an almost universal option for this type of study. Generally, it precedes the application of supervised methods that allow sample discrimination and assignment to pre-established classes. Complementary information on the most descriptive biomarkers or class signals is obtained as well. Linear discriminant analysis and partial least squares-discriminant analysis have provided excellent results in differentiating botanical varieties and geographical origins of cocoa samples. Finally, multivariate calibration methods, especially partial least square regression, make it possible to quantitate complex features such as solid contents, cocoa butter equivalents, or percentages of adulterants, such as cocoa shells and other substances added to increase the product mass.

Fingerprinting (or untargeted analysis) is currently the most widespread option to address food characterization, classification, and authentication issues. The great success of this approach relies on its simplicity; besides, no preliminary information or preconceived ideas about the nature of the analytes are required. If a given fingerprint contains discriminant features, chemometric methods detect and exploit them to establish differentiation, classification, or quantification models of adulterations. IR spectroscopies (near and medium), NMR, and MS are the most popular techniques for cocoa and chocolate analyses, as summarized in Table 1 and Table 2. Similarly, untargeted chromatography (with UV, fluorescence, or MS detection), based on a general separation gradient, is another powerful option with excellent analytical performance. In any case, complex signals related to the composition of the samples are generated and analyzed, often chemometrically. Unfortunately, the joint study of fingerprints obtained on different days or sessions is usually complex since variations in sensitivity, instrumental drifts, and peak shifting are difficult to correct. Therefore, the pre-treatment and standardization of the data are essential in fingerprinting. Although elucidating the corresponding (known or unknown) chemical descriptors responsible for those relevant signals is unnecessary, the knowledge of the associated biomarkers can be very enriching, leading to an increased knowledge about which discriminant compounds can be used in further targeted analysis. Portable and handheld devices to analyze the samples in situ and obtain results in real-time on the sample quality and authenticity is also a fundamental issue within untargeted approaches. Currently, simple, and cheap commercial spectrophotometers are available for robust and rapid UV-visible, IR, and Raman spectral measurements. This low-cost equipment will also play a key role in areas and circumstances where more expensive equipment cannot be afforded, so all applications and innovations in this regard will be very welcome.

The targeted approach has some pros and cons compared to the untargeted counterpart. In our view, the greatest advantage of profiling is that the concentrations determined by validated methods are accurate, precise, and robust. Hence, the comparison and simultaneous study of compositional data obtained from different batches, working sessions, instruments, and laboratories is not a dramatic problem. This aspect is very relevant to deal with authentication routinely, without the need to analyze the calibration samples and create mathematical models each time. Some drawbacks of this option, apart from the need to have previous information about discriminant components, are that the calibration can be time-consuming and more experimentally complex, and analyte standards can be expensive. As detailed in Table 3 and Figure 1, the polyphenolic composition is the preferred option to address authentication studies. Due to their joint availability, theobromine and caffeine concentrations are often analyzed with polyphenols. Other families of analytes also seem to be markers with remarkable discriminating power, emphasizing the amino compounds and the volatiles. Inorganic information also appears to be important, especially for traceability of origin.

In short, the issue of cocoa authentication is complex and exciting. The most convenient analytical strategies to carry it out are developed here. The bibliography reveals the growing interest that it arouses, with almost exponential growth in the number of annual publications and citations this topic receives.

## Data Availability

Data is contained within the article.
